# Multifunctional Interleukin-24 Resolves Neuroretina Autoimmunity via Diverse Mechanisms

**DOI:** 10.3390/ijms231911988

**Published:** 2022-10-09

**Authors:** Xuan Zhang, Cuiping Hu, Yajie Zhong, Dijie Qiao, Wei Chi, Huangxuan Shen, Waipo Chong

**Affiliations:** 1 State Key Laboratory of Ophthalmology, Zhongshan Ophthalmic Center, Sun Yat-sen University, Guangzhou 510060, China; 2 School of Chinese Medicine, Hong Kong Baptist University, Hong Kong SAR, China

**Keywords:** IL-24, T helper cells, RPE cells, autoimmune uveitis

## Abstract

IL-24 is a multifunctional cytokine that regulates both immune cells and epithelial cells. Although its elevation is associated with a number of autoimmune diseases, its tolerogenic properties against autoreactive T cells have recently been revealed in an animal model of central nervous system (CNS) autoimmunity by inhibiting the pathogenic Th17 response. To explore the potential of IL-24 as a therapeutic agent in CNS autoimmunity, we induced experimental autoimmune uveitis (EAU) in wildtype mice and intravitreally injected IL-24 into the inflamed eye after disease onset. We found that the progression of ocular inflammation was significantly inhibited in the IL-24-treated eye when compared to the control eye. More importantly, IL-24 treatment suppressed cytokine production from ocular-infiltrating, pathogenic Th1 and Th17 cells. In vitro experiments confirmed that IL-24 suppressed both Th1 and Th17 differentiation by regulating their master transcription factors T-bet and RORγt, respectively. In addition, we found that intravitreal injection of IL-24 suppressed the production of proinflammatory cytokines and chemokines from the retinas of the EAU-inflamed eyes. This observation appears to be applicable in humans, as IL-24 similarly inhibits human retinal pigment epithelium cells ARPE-19. In conclusion, we report here that IL-24, as a multifunctional cytokine, is capable of resolving ocular inflammation in EAU mice by targeting both uveitogenic T cells and RPE cells. This study sheds new light on IL-24 as a potential therapeutic candidate for autoimmune uveitis.

## 1. Introduction

Autoimmune uveitis represents a group of ocular inflammatory diseases that are caused by the autoimmunity targeting the antigens in the retina and/or choroid. It leads to ocular inflammation, neural retinal damage, and ultimately, vision loss [1]. In an animal model of experimental autoimmune uveitis (EAU), the autoimmune response against the retina is initiated by both T helper 1 (Th1) and Th17 cells, which target the retinal–antigen interphotoreceptor retinoid-binding protein (IRBP) [2,3]. Upon activation, these cells migrate to the retina and trigger ocular inflammation [1,4,5]. In healthy individuals, the eyes are protected from inflammation to avoid vision loss. This ocular immune privilege requires the retinal pigment epithelium (RPE) cells to maintain the integrity of the blood–retina barrier (BRB) [6]. Under the influence of ocular infiltrating uveitogenic T cells, the RPE cells can become pathogenic and express different proinflammatory molecules, which leads to the breakdown of BRB and retinal damage [7]. Therefore, a better therapeutic approach should have the capacity to target both uveitogenic T cells and RPE cells.

Interleukin (IL)-24 belongs to the IL-10 family. Together with IL-19 and IL-20, these are known as IL-20 receptor cytokines because their receptors are comprised of the IL-20 receptor β-subunit (IL-20RB) [8]. IL-24 has two heterodimeric receptors, i.e., IL-20RB, with either the IL-20 receptor α-subunit (IL-20RA) or the IL-22 receptor α1-subunit (IL-22RA1) [9]. IL-24 and its receptors are widely expressed by immune cells and epithelial cells, and IL-24 elevation is associated with different autoimmune diseases, including psoriasis [10], rheumatoid arthritis [11], inflammatory bowel diseases [12], and multiple sclerosis [13], suggesting that it is a multifunctional cytokine that may play a critical role in regulating tissue inflammation and autoimmunity.

IL-24 is produced by different immune cells, including T cells, B cells, natural killer (NK) cells, monocytes, and macrophages [9]. It has been shown to be a Th2 cytokine [14], inhibiting the production of interferon (IFN)-γ and IL-17A from Th1 and Th17 cells, respectively [15,16]. Our recent study revealed that not only Th2 cells, but also Th17 cells produce this inhibitory cytokine [17]. Upon stimulation, Th17 cells produce the signature cytokine, IL-17A, which has a negative feedback to Th17 cells for induction of IL-24, subsequently suppressing other proinflammatory cytokines [17]. Mechanistically, IL-24 suppresses production of those proinflammatory cytokines by inducing the expression of suppressors of cytokine-signaling proteins [17]. However, its roles in regulating Th1 and Th17 cell differentiation have not been well characterized.

Despite its regulatory properties affecting T-cell functions, IL-24 is susceptible to some autoimmune diseases. Its elevation is associated with disease pathogenesis in animal models of psoriasis and stimulates the expression of proinflammatory molecules from human keratinocytes, including IL-20, CXCL1, CXCL8, CCL20, psoriasin, and LCN2 [18]. Blocking of the IL-20 receptor ameliorates collagen-induced arthritis [19] and stimulates synovial fluid mononuclear cells to produce CCL2 for further cellular infiltration [11]. However, our recent study highlighted the tolerogenic properties of IL-24 in regulating CNS autoimmune diseases, showing that mice deficient in IL-24 were more susceptible to EAU and experimental autoimmune encephalomyelitis (EAE) [17]. Therefore, we hypothesized that IL-24 has a complex role in regulating different autoimmune diseases and may potentially suppress autoimmune uveitis by regulating autoreactive T cells and RPE cells.

In this study, we found that intravitreal injection of recombinant IL-24 after disease onset significantly inhibited the development of EAU. Mechanistically, IL-24 treatment reduced pathogenic Th1 and Th17 responses in the inflamed eye and suppressed the production of proinflammatory molecules by the retina. This suppression appears to be applicable in humans, as IL-24-treated human RPE cells also expressed significantly lower levels of proinflammatory cytokines and chemokines.

## 2. Results

### 2.1. IL-24 Inhibits Differentiation of Th1 and Th17 Cells and Their Production of Proinflammatory Cytokines

**Figure 1:**
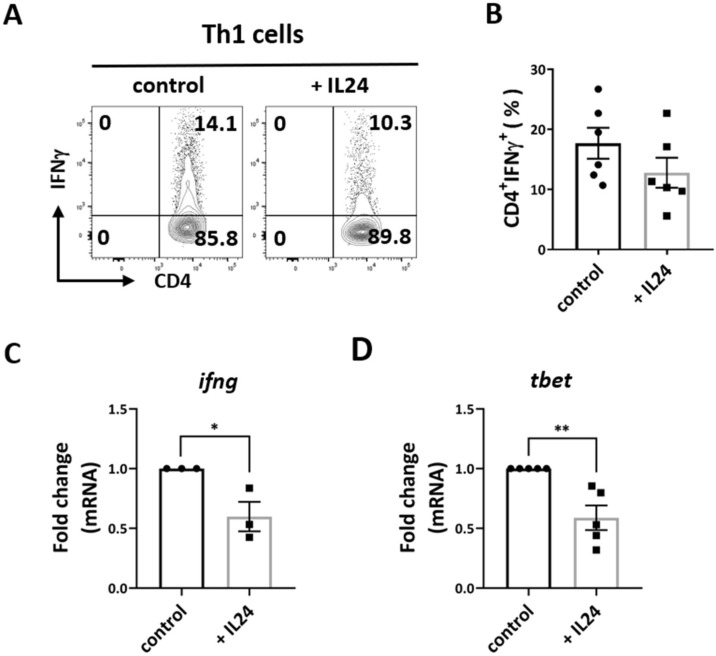
CD4^+^CD62L^+^ T cells from spleens and LNs of C57BL/6 mice were isolated and polarized under Th1 conditions with anti-CD3/CD28 antibodies for 3 days, with and without recombinant IL-24. (**A**,**B**) Representative FCM plots (**A**) and compiled data (**B**) of IFN-γ expression in Th1 cells. Data represent means ± SEMs of six independent experiments. (**C**,**D**) The relative gene expression of ifng and tbet in Th1 cells. Data represent the means ± SEMs of three (**C**) or five (**D**) independent experiments. * *p* < 0.05, ** *p* < 0.01, Student’s *t* test.

We and others have previously reported that IL-24 inhibits the production of proinflammatory cytokines from Th1 and Th17 cells in cases of inflammatory and infectious disease [15,16,17]. To investigate whether IL-24 could regulate Th1 and Th17 cell differentiation directly, we polarized naive CD4^+^ T cells under Th1 and Th17 polarization conditions with recombinant IL-24. First, we found that IL-24 significantly suppressed Th1 expression of IFN-γ (Figure 1A–C). IFN-γ is the signature cytokine of Th1 cells promoting inflammation and activates other immune cells, including cytotoxic CD8^+^ T cells and NK cells, to produce IFN-γ as well [20]. Second, we found that IL-24 significantly suppressed the expression of T-bet (Figure 1D), which is the master transcription factor for Th1 cells for initiating the differentiation of naive CD4^+^ T cells into Th1 cells. These data suggest that IL-24 negatively regulates both the effector function and the differentiation of Th1 cells.

**Figure 2:**
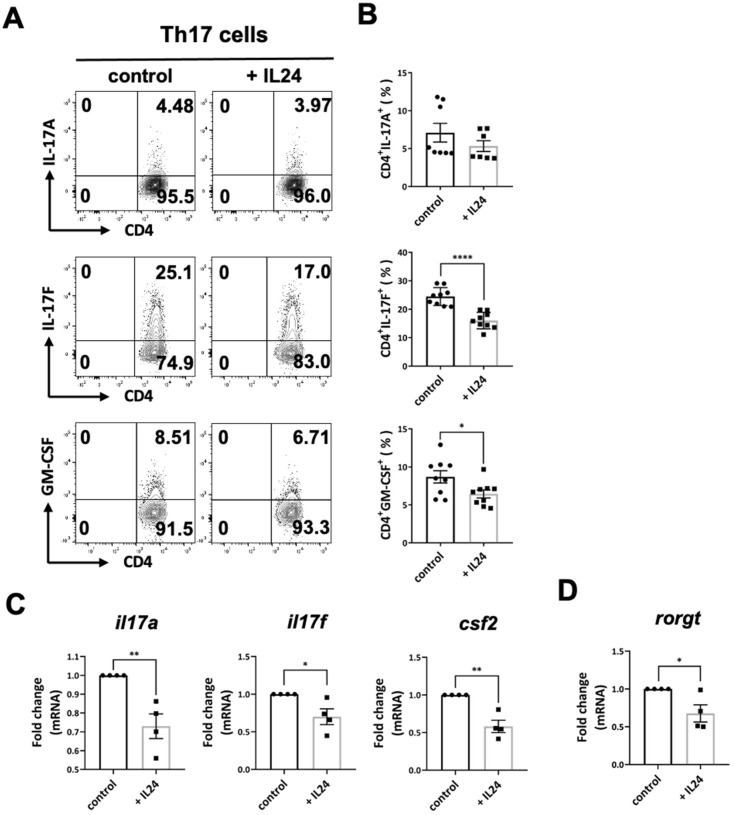
IL-24 inhibited Th17 cells. CD4^+^CD62L^+^ T cells from spleens and LNs of C57BL/6 mice were isolated and polarized under Th17 conditions with anti-CD3/CD28 antibodies for 3 days, with and without recombinant IL-24. (**A**,**B**) Representative FCM plots (**A**) and compiled data (**B**) of IL-17A, IL-17F, and GM-CSF expression in Th17 cells. Data represent means ± SEMs of three independent experiments. * *p* < 0.05, **** *p* < 0.0001, Student’s *t* test. (**C**,**D**) The relative gene expression of IL17A, IL17F, CSF2, and RORγt in Th17 cells. Data represent means ± SEMs of 4 independent experiments. * *p* < 0.05, ** *p* < 0.01, Student’s *t* test.

Th17 cells are the other lineage of effector CD4^+^ T cells that drive autoimmunity [21]. They express high levels of proinflammatory cytokines, such as IL-17A, IL-17F, and GM-CSF, resulting in tissue damage and neutrophil recruitment [21]. As in our previous study [17], we showed that IL-24 significantly suppressed Th17 cell production of IL-17F and GM-CSF (Figure 2A–C). We found that IL-24 could not suppress IL-17A at the protein level, but reduced the gene expression of IL-17A. The expression of the master transcript factor of Th17 cells, RORγt, was also downregulated by IL-24 (Figure 2D), suggesting that, similar to Th1 cells, IL-24 regulates the expression of proinflammatory cytokines from Th17 cells, as well their differentiation.

### 2.2. IL-24 Does Not Induce the Expression of Immunoregulatory IL-10 or the Differentiation of Regulatory T Cells

A deficiency in IL20RB, the beta-subunit of the IL-24 receptor, is associated with decreased IL-10 expression in animals [22]. Therefore, we hypothesized that IL-24 may induce Th1 and Th17 cells to express IL-10 for immunoregulation. However, we found that IL-24 did not induce IL-10 expression by these cells (Supplementary Figure S1A).

In addition to differentiating into effector cells, naive CD4^+^ T cells can differentiate into immunosuppressive cells, i.e., regulatory T (Treg) cells. Therefore, we also investigated the effect of IL-24 on Treg cell differentiation. We polarized naive CD4^+^ T cells into Treg cells with and without recombinant IL-24 and determined the expression of the master transcription factor for Treg cells, Foxp3, using flow cytometry. We found that recombinant IL-24 did not alter Foxp3 expression (Supplementary Figure S1B), suggesting that IL-24 does not affect Treg cell differentiation.

### 2.3. Intravitreal Injection of IL-24 Resolves Ocular Inflammation and Suppresses Proinflammatory Cytokine Production from Ocular-Infiltrating CD4^+^ T Cells

**Figure 3:**
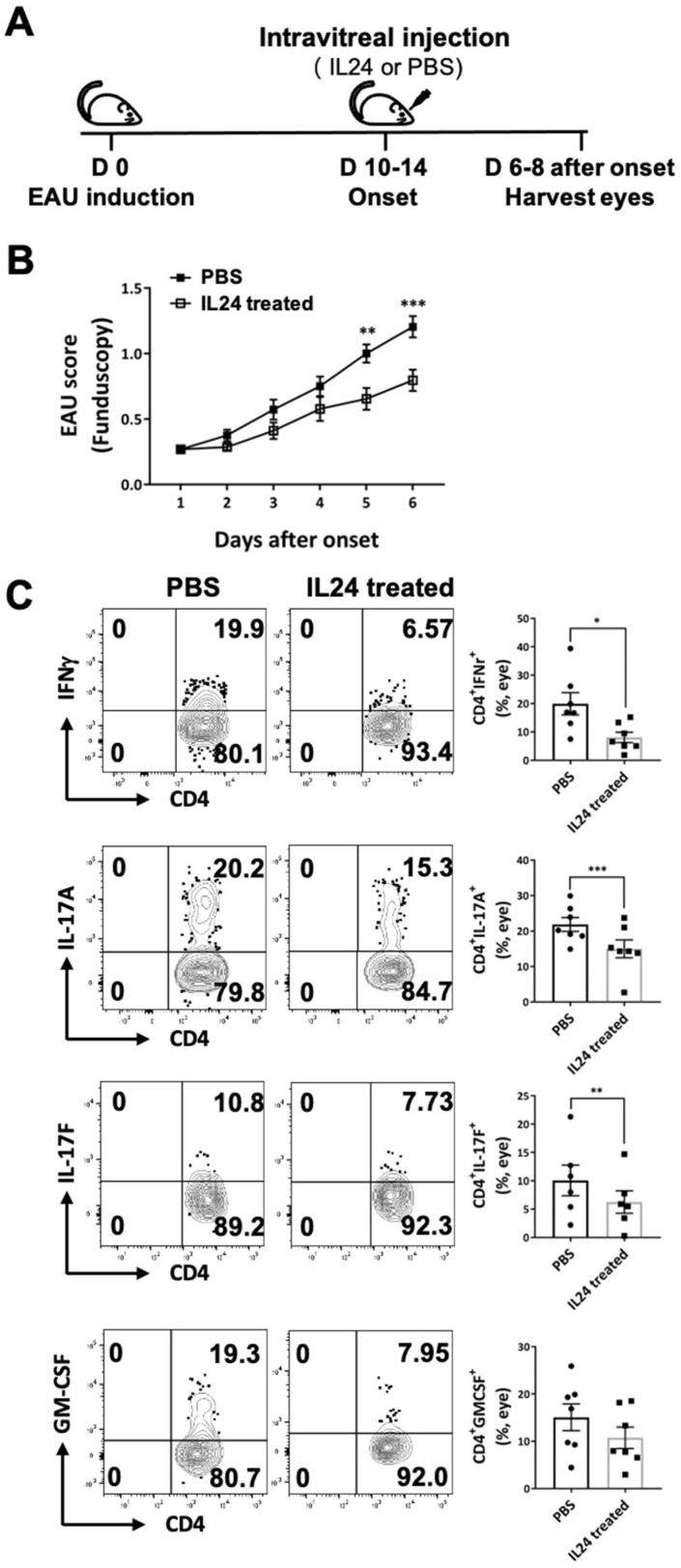
IL-24 suppressed ocular inflammation in EAU mice. EAU was induced in C57BL/6 mice by active immunization with IRBP_1-20_ and CFA. (**A**) Each of the eyes was injected intravitreally with either recombinant IL-24 or PBS on the day of disease onset. Eyes were harvested on day 6-8 after onset. (**B**) EAU disease score was determined by funduscopy. Eyes treated with recombinant IL-24 developed significantly lower disease than the controls. Compiled data from three independent experiments, means ± SEMs (PBS: n = 14 eyes, IL-24 treated: n = 14 eyes). ** *p* < 0.05, *** *p* < 0.001, Two-way ANOVA. (**C**) Representative FCM plot (left) and compiled data (right) of IFN-γ, IL-17A, IL-17F, and GM-CSF production of ocular-infiltrating CD4^+^ T cells on day 6–8 after onset. Compiled data from two independent experiments (PBS: n = 7 eyes, IL-24 treated: n = 7 eyes). Means ± SEMs, * *p* < 0.05, ** *p* < 0.05, *** *p* < 0.001, paired *t*-test.

We previously reported that IL-24^−/−^ mice were less susceptible to EAE and EAU [17], suggesting that IL-24 may serve as a novel therapeutic treatment for T cell-mediated autoimmune diseases. To test this hypothesis, we intravitreally injected IL-24 into the inflamed eye of each EAU mouse on the day of disease onset (10 to 14 days after EAU immunization), with the other eye receiving PBS as a negative control (Figure 3A). We monitored disease development using fundoscopy and found that IL-24 significantly suppressed the development of ocular inflammation, while inflammation continued to progress in the eyes that received PBS (Figure 3B).

After 6–8 days of intravitreal injection treatment, we harvested the eyes and studied the cytokine profile of the ocular-infiltrating CD4^+^ T cells using intracellular cytokine staining. We found that the T cells isolated from the IL-24-treated eyes expressed significantly lower levels of Th1 and Th17 cytokines, including IFN-γ, IL-17A, IL-17F, and GM-CSF (Figure 3C). These outcomes suggest that IL-24 treatment inhibits pathogenic Th1 and Th17 cells and protects the eyes from ocular inflammation.

### 2.4. IL-24 Inhibits Peripheral Antigen-Specific Response

We showed that intravitreal injection of IL-24 into inflamed eyes significantly inhibits tissue inflammation and ocular-infiltrating CD4^+^ T cell expression of proinflammatory cytokines. We next investigated whether IL-24 could also inhibit the peripheral uveitogenic (IRBP-specific) T-cell response. To study this, we isolated the splenocytes of EAU mice and restimulated uveitogenic T cells with anti-CD3/28 antibodies and IRBP_1-20_ for 48 h with and without recombinant IL-24. We found that recombinant IL-24 significantly reduced IRBP_1-20_-specific IFN-γ production, but could not suppress IL-17A, IL-17F, or GM-CSF production (Supplementary Figure S2). These data suggest that IL-24 is capable of suppressing the peripheral uveitogenic response in Th1, but not in Th17.

### 2.5. IL-24 Reduces the Expression of Proinflammatory Molecules by the Retina in EAU Mice

**Figure 4:**
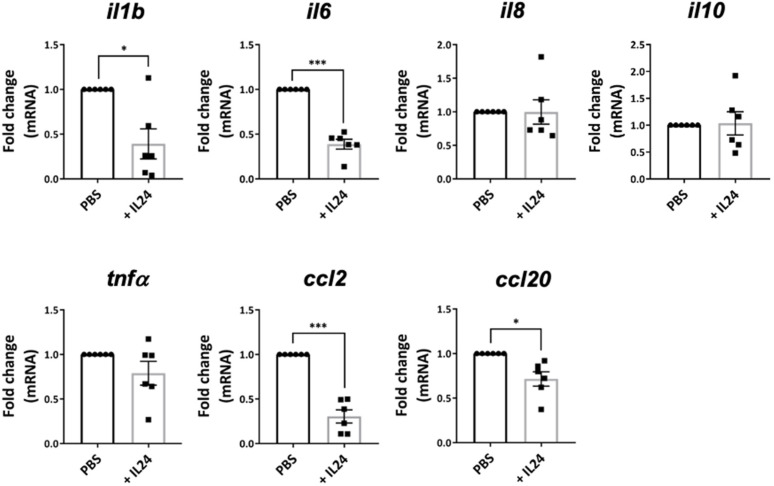
IL-24 suppressed the production of proinflammatory molecules in the retina. EAU was induced in C57BL/6 mice by active immunization with IRBP_1-20_ and CFA. Each of the eyes was injected intravitreally with either recombinant IL-24 or PBS on the day of disease onset. The eyes were harvested 6–8 days after treatment. Retinas were isolated for cDNA synthesis. Relative gene expressions of *il1b*, *il6*, *il8*, *il10*, *tnfa*, *ccl2,* and *ccl20* were quantified by qPCR. Compiled data from two independent experiments (PBS: n = 6 eyes, IL-24-treated: n = 6 eyes). Means ± SEMs, * *p* < 0.05, *** *p* < 0.001, paired *t* test.

IL-24 has been demonstrated to regulate tissue inflammation via IL-24 receptor expression in epithelial cells, such as in keratinocytes in psoriasis and colonic epithelial cells in inflammatory bowel disease [9]. Therefore, we studied the effect of IL-24 on the expression of proinflammatory molecules in the retinas of IL-24-treated, inflamed eyes of EAU mice. We harvested the retinas on day 6 after intravitreal injection of recombinant IL-24 or PBS. The retina was isolated in the manner previously described [23], and mRNA was extracted for cDNA synthesis. Complementing our observation that IL-24 treatment inhibited the progression of ocular inflammation in EAU mice (Figure 3B), we found that IL-24 also significantly inhibited the gene expression of IL-1β, IL-6, CCL2 and CCL20 (Figure 4).

### 2.6. IL-24 Inhibits the Production of Proinflammatory Molecules by Human RPE Cells

**Figure 5:**
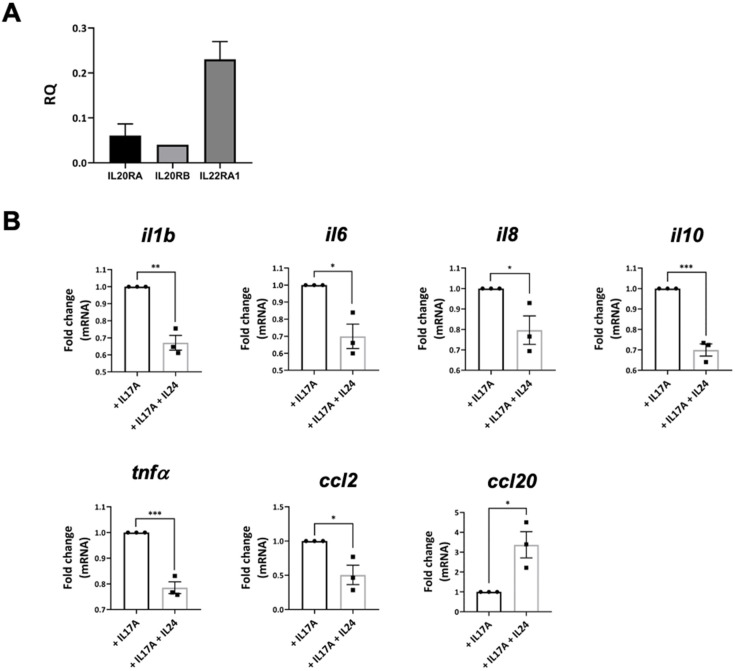
IL-24 inhibited the production of inflammatory cytokines in ARPE-19 cells. (**A**) Gene expression of IL-24 receptor in ARPE-19 cells was determined by qPCR. (**B**) ARPE-19 cells were stimulated by recombinant IL-17A with and without recombinant IL-24. After 24 h of incubation, the relative gene expressions of *il1b*, *il6*, *il8*, *il10*, *tnfa*, *ccl2,* and *ccl20* were measured by qPCR. Data represent means ± SEMs of three independent experiments. * *p* < 0.05, ** *p* < 0.01, *** *p* < 0.001, Student’s *t* test.

RPE cells play an important role in maintaining ocular immune privilege by expressing immunosuppressive molecules [6]. However, these RPE cells can become proinflammatory upon stimulation by various stress signals, such as IL-17A, and release proinflammatory cytokines and chemokines, leading to retina inflammation and damage. Here, we showed that IL-24 inhibited the expression of proinflammatory molecules in the retinas of EAU mice (Figure 4). To investigate whether IL-24 could also regulate human RPE cells, we first studied the expression of IL-24 receptors. IL-24 signal through two heterodimeric receptors, i.e., IL-20RB/IL-20RA and IL-20RB/IL-22RA1 [9]. We found that the human RPE cell line, ARPE-19, expressed high levels of IL-20RA and IL-22RA1 (Figure 5A). A relatively moderate expression level of the common subunit IL-20RB was also observed (Figure 5A).

We next used IL-17A to stimulate ARPE-19 cells with and without IL-24. Upon IL-17A stimulation, ARPE cells expressed high levels of proinflammatory molecules, including IL-1β, IL-6, IL-8, TNF-α, CCL2 and CCL20 (Figure 5B). We found that adding IL-24 significantly suppressed the gene expression of those proinflammatory molecules, except CCL20 (Figure 5B). Interestingly, IL-17A also stimulated ARPE cells to express the immunosuppressive cytokine IL-10, which is downregulated by IL-24 (Figure 5B). These data suggest that IL-24 inhibits the production of proinflammatory molecules by human RPE cells.

## 3. Discussion

IL-24 is a multifunctional cytokine that regulates both immune cells and epithelial cells [9]. We recently identified its role in limiting Th17 pathogenicity in CNS autoimmunity by suppressing proinflammatory cytokines from Th17 cells [17]. However, IL-24′s potential as a new therapeutic candidate for treating autoimmune diseases has not been fully explored. In this study, we found that intravitreal injection of IL-24 into the inflamed eyes of EAU mice significantly inhibited the progression of ocular inflammation. More importantly, it suppressed the expression of proinflammatory cytokines from ocular-infiltrating Th1 and Th17 cells. Mechanistic studies confirmed that IL-24 inhibits Th1 and Th17 cell differentiation and cytokine production by targeting the master transcription factors of Th1 and Th17 cells (T-bet and RORγt, respectively). Additionally, IL-24 treatment significantly reduced the expression of proinflammatory molecules by the retinas of inflamed eyes. This may also apply to human eyes as well, as IL-24 also downregulated those proinflammatory molecules in human RPE cells.

Systemic immunosuppression is widely used to treat autoimmune uveitis. Conventional immunosuppressants include antimetabolites (methotrexate, mycophenolate, azathioprine), calcineurin inhibitors (cyclosporine, tacrolimus), and alkylating agents (cyclophosphamide and chlorambucil) [24]. Additional biologics, such as inhibitors of proinflammatory cytokines, should also be considered if the conventional immunosuppressants are not effective in certain patients [24]. However, long-term systemic immunosuppression leads to susceptibility to infectious diseases or even malignancy. Thus, local treatment may present a better option by providing a precise approach to drug delivery to the site of inflammation, with fewer adverse effects. Our recent study reported that systemic depletion of IL-24 exacerbated the development of EAU and systemic administration of high doses of recombinant IL-24 ameliorated EAU [17]. However, the elevation of IL-24 is also associated with other autoimmune diseases, including psoriasis, rheumatoid arthritis, and inflammatory bowel disease. This suggests that IL-24 may be pathogenic in other types of tissue inflammation, not making it an ideal candidate for the systemic treatment of autoimmune uveitis, which is usually a manifestation of active systemic autoimmune diseases [25]. Therefore, we intravitreally injected recombinant IL-24 into the inflamed eyes of EAU mice and demonstrated that it is capable of suppressing ocular inflammation, supporting its potential as a local treatment for patients with autoimmune uveitis. In order to better evaluate IL-24 as suitable as a long-term treatment for autoimmune uveitis, future studies in EAU should be carried out with longer observation and multiple doses of IL-24 injection. ERG and OCT imaging should also be included to better examine the therapeutic effect of IL-24.

IL-24 has recently been identified as an inhibitory cytokine that regulates Th1 and Th17 responses [9]. The T cells of patients with lymphatic filariasis or pulmonary tuberculosis expressed high levels of IL-24, suppressing Th1 and Th17 cells into producing their signature cytokines, IFN-γ and IL-17A, respectively [15,16]. In another study with CD4^+^ T cells from healthy individuals, IL-24 dampened their expression of IFN-γ after stimulation with anti-CD3/CD28 antibodies [26] and suppressed the expression of Th17-related cytokines from Th17 cells [17]. In this study, we found that IL-24 not only inhibited the production of cytokines from T cells but, more importantly, abrogated the differentiation of naive CD4^+^ T cells into effector Th1 and Th17 cells by downregulating the master transcription factors T-bet and RORγt. Cytokine milieu is critical for the induction of T-bet and RORγt, as it activates the Janus family kinases (JAK)/signal transducer and activates transcription (STAT) signaling. For Th1 cells, IL-12 and IFN-γ are responsible for inducing T-bet through the phosphorylation of STAT4 and STAT1, respectively, which initiates the Th1 differentiation program [27,28]. In the case of Th17 cells, IL-6 and IL-23 signaling promotes the phosphorylation of STAT3, which then migrates to the nucleus and promotes the expression of RORγt, the master regulator of Th17 cells [29]. IL-24 belongs to the IL-20R cytokine and its signaling is also mediated through STAT1 and STAT3 [30]. Therefore, IL-24 may be involved in the regulation of STAT1/T-bet and STAT3/RORγt in Th1 and Th17 cells, respectively. Future studies will be required to reveal the molecular mechanism by which IL-24 regulates the differentiation of Th1 and Th17 cells.

We found that intravitreal injection of IL-24 also inhibited the ability of the retinas of EAU mice to produce proinflammatory molecules. Within the retina, RPE cells are critical for maintaining eye immune privilege, as they preserve the BRB and produce immunoregulatory molecules. They also express IL-20R and have been shown to be activated by IL-22, one of the IL-20R cytokines, to produce the proinflammatory chemokine CCL2 [31]. Unlike IL-22, we found that IL-24 negatively regulated the production of proinflammatory molecules from human RPE cell lines (ARPE-19). These observations support the notion that IL-24 could resolve ocular inflammation by regulating RPE cells. Interestingly, IL-22 has also been shown to induce the apoptosis of RPE cells [32]. Follow-up studies will be required to clarify the role of IL-24 in regulating RPE cell differentiation, proliferation, and apoptosis.

CCL20 is mainly produced by epithelial cells, and its expression is increased in the inflammatory environment. Previous studies have shown that CCL20-CCR6 plays an important role in a variety of autoimmune diseases, such as RA, IBD, and other diseases, and the level of CCL20 is associated with disease severity [33], as well as in EAU [34]. In accordance to previous observations, our data showed that IL-24 treatment significantly inhibited the expression of CCL20 expression in the retina of EAU mice. However, it increased the level of CCL20 in IL-17A-stimulated ARPE cells. This inconsistency may be due to the different in the experimental setup because IL-17A stimulation is not enough to represent the proinflammatory environment in uveitis for the stimulation of ARPE-19.

In vitro studies showed that IL-24 significantly suppressed IFN-γ and IL-17A production from Th1 and Th17 cells (Figure 1 and Figure 2). However, it only diminished IFN-γ production in the IRBP stimulated splenocytes (Supplementary Figure S2). Vitreous cavity-associated immune deviation (VCAID) may be one of the reasons for this observation. VCAID represents abnormal systemic immune response induced by antigen placed in vitreous cavity of eyes. However, previous studies have shown that VCAID can be eliminated by vitreous inflammation such as EAU like ACAID [35]. Future studies should be carried out to better clarify these observations.

In conclusion, we have demonstrated that intravitreal injection of recombinant IL-24 can resolve ocular inflammation in EAU mice by inhibiting the production of proinflammatory cytokines and chemokines from ocular-infiltrating Th1 and Th17 cells and the RPE cells. In vitro studies revealed that IL-24 can suppress the expression of T-bet and RORγt, the master regulators of Th1 and Th17 cells, respectively. This study highlights the potential of IL-24 as a promising therapeutic agent for autoimmune uveitis.

## 4. Materials and Methods

### 4.1. Mice

Wildtype C57BL/6 mice were purchased from the Guangzhou Animal Experiment Company. Mice were housed in a pathogen-free facility. The animals were handled and cared for according to the Association for Research in Vision and Ophthalmology guidelines for use of animals in ophthalmic and vision research and with the approval of the Animal Care and Use Committee of the Zhongshan Ophthalmic Center, Sun Yat-sen University (ref no. 2021-025).

### 4.2. ARPE-19 Cell Cultures

A human retinal pigment epithelial cell line (ARPE-19) was obtained from the American Type Culture Collection (ATCC, Manassas, VA, USA). ARPE-19 cells were cultured in DMEM: F-12 Medium (ATCC, Manassas, VA, USA) with 10% fetal bovine serum (FBS, GIBCO, Grand Island, NY, USA) and 1% penicillin/streptomycin in a humidified incubator at 37 °C and 5% CO_2_. Cells were passaged in a ratio of 1:3 when density reached 80%–90%, and the media were changed twice weekly. Passages between 10 and 15 were used for experiments.

### 4.3. Induction, Treatment, and Evaluation of EAU

To induce EAU, C57BL/6 mice were immunized with a 200 µL emulsion of 150 µg human IRBP_1–20_ (GPTHLFQPSLVLDMAKVLLD, Hanhong, China) in an equal volume of complete Freund’s adjuvant (CFA, Sigma-Aldrich, St. Louis, MO, USA) containing 2.5 mg/mL Mycobacterium tuberculosis (strain 37RA) (Sigma-Aldrich, St. Louis, MO, USA) and 0.5 µg of *Bordetella pertussis* toxin (PTX, Sigma-Aldrich, St. Louis, MO, USA), as described in a previous study [36].

For treatment, EAU mice were intravitreally injected with mouse-recombinant IL-24 (200 ng in 2 µL) (R&D Systems, Minneapolis, MN, USA) into the left eye at the onset of disease. As a negative control, 2 µL of PBS was injected into the right eye. Eyes were examined by funduscopy at regular intervals using the Micron-IV retinal imaging system for small animals (Phoenix Research Laboratories, Pleasanton, CA, USA) and scored on a scale of 0–4, as described in previous study [37]. Experiments were terminated on day 6–8 after disease onset for eye collection.

### 4.4. Isolation of Infiltrating Cells from Eyes

After the mice were sacrificed at the indicated time points, their eyes were carefully isolated and dissected and the lenses removed. The remaining tissues were fully minced on ice and then incubated for 50 min with 1 mg/mL collagenase D (Sigma-Aldrich, St. Louis, MO, USA) in RPMI (GIBCO, Grand Island, NY, USA) containing 10% FBS at 37 °C. After incubation, the isolated cells were washed, filtered, and re-suspended in RPMI containing 10% FBS for future intracellular staining.

### 4.5. Intracellular Cytokine Staining for FCM Analysis

Single-cell suspensions were prepared as described above. Cells were stimulated for 4 h with 50 ng/mL of PMA (Sigma-Aldrich, St. Louis, MO, USA) and 500 mg/mL of ionomycin (Sigma-Aldrich, St. Louis, MO, USA) in the presence of GolgiStop (BD Biosciences, San Diego, CA, USA). After stimulation, the cells were washed and resuspended in a staining buffer, where they were stained with anti-mouse CD3 (145-2C11, BD Bioscience, San Jose, CA, USA), CD4 (GK1.5, Biolegend, San Diego, CA, USA), and CD8 (53-6.7, BD Bioscience, San Jose, CA, USA) antibodies and incubated for 30 min at 4 °C in PBS with 2% FBS. Cells were then fixed and permeabilized with the Cytofix/Cytoperm Solution Kit (BD Biosciences, San Jose, CA, USA) and stained with anti-mouse IFN-γ (XMG1.2, Biolegend, San Diego, CA, USA), IL-17A (TC11-18H10, Biolegend, San Diego, CA, USA), IL-17F (O79-289, Biolegend, San Diego, CA, USA), and GM-CSF (MP1-22E9, Biolegend, San Diego, CA, USA) antibodies for 1 h at room temperature. The results were analyzed using a BD LSRFortessa Cell Analyzer and BD FlowJo (Tree Star Inc., Oakland, CA, USA).

### 4.6. T-cell Activation and Differentiation

Cells were isolated from spleens and draining lymphoid nodes (dLNs) of EAU mice on day 8–10 post-onset. Single-cell suspensions were prepared as described above. Cells were stimulated with IRBP_1–20_ peptide at a density of 2.5 × 10^6^ cells/mL with and without IL-24 (50 ng/mL) in 96-well U-bottom plates for 48 h at 37 °C. Cytokines were measured from the cultured supernatants using ELISA: IFN-γ, IL-17A, GM-CSF (Invitrogen, San Diego, CA, USA), and IL-17F (Multisciences, Zhejiang, China).

CD4^+^CD62L^+^ T cells were isolated from spleens and lymph nodes (LNs) using a cell sorter (FACSAriaIII Fusion, BD Biosciences, San Diego, CA, USA). Cells were stimulated with 2 µg/mL plate-bound anti-CD3 (145-2C11; Bio X Cell, Lebanon, NH, USA) and 1 µg/mL soluble anti-CD28 (37.51; Bio X Cell, Lebanon, NH, USA) antibodies. For Th17 polarization, culture media were supplemented with 30 ng/mL IL-6, 2.5 ng/mL TGF-β (R&D Systems, Minneapolis, MN, USA), 10 µg/mL anti-IFN-γ, and 10 µg/mL anti-IL-4 (eBioscience, San Diego, CA, USA) antibodies. For Th1 polarization, culture media were supplemented with 10 ng/mL IL-12 (Biolegend, San Diego, CA, USA) and 10 µg/mL IL-4 (eBioscience, San Diego, CA, USA) antibodies. For Treg polarization, culture media were supplemented with 5 ng/mL TGF-β (R&D Systems, Minneapolis, MN, USA) antibodies. For experimental groups, 50 or100 ng/mL mouse-recombinant IL-24 was added. On day 3, cells were pulsed with PMA and ionomycin in the presence of GolgiStop for 4 h. Cytokine expression was determined with intracellular cytokine staining and real-time PCR.

### 4.7. Real-Time PCR

Next, 5 × 10^5^ of ARPE-19 cells were seeded in 6-well plates in DMEM: F-12 medium containing 10% FBS and allowed to adhere overnight. The media were changed to DMEM: F-12 without FBS and cultured for 24 h before being changed to DMEM: F-12 containing 10% FBS with or without 10 ng/mL human-recombinant IL-24 (Sino Biological, Beijing, China) for 24 h. The media were removed and cells were washed twice with PBS. Total RNA was extracted using RNAiso Plus reagent (Takara, Tokyo, Japan) and cDNA was synthesized using High-FastKing gDNA Dispelling RT SuperMix Kit (Tiangen, Beijing, China), following manufacturers’ instructions. Targeted gene expression was detected using the ABI StepOnePlus system. Relative gene expression was determined with a 2^−ΔΔCT^ formula. The primers for qPCR were purchased from Thermo Fisher Scientific and are listed in Table 1.

### 4.8. Statistical Analysis

All data were expressed as mean ± SEM and statistical analyses were performed using Graphpad Prism 8.0 (GraphPad Software, Inc., La Jolla, CA, USA). For statistical analysis, paired or unpaired *t*-tests were used for two-group comparisons, a one-way ANOVA or two-way ANOVA was used for multi-group comparisons, and a *p*-value of less than 0.05 was interpreted as significant.

## Figures and Tables

**Table 1:** Primer information.

**Human Genes**		**Primer (5′ to 3′)**
*gapdh*	Forward	ACAACAGCCTCAAGATCATCAG
	Reverse	TCTTCTGGGTGGCAGTGATG
*il6*	Forward	TCTGCGCAGCTTTAAGGAGT
	Reverse	CCCAGTGGACAGGTTTCTGA
*tnfa*	Forward	GAGGCCAAGCCCTGGTATG
	Reverse	CGGGCCGATTGATCTCAGC
*ccl2*	Forward	GCTCATAGCAGCCACCTTCATTC
	Reverse	GTCTTCGGAGTTTGGGTTTGC
*il8*	Forward	ACTGAGAGTGATTGAGAGTGGAC
	Reverse	AACCCTCTGCACCCAGTTTTC
*il1b*	Forward	TTCGACACATGGGATAACGAGG
	Reverse	TTTTTGCTGTGAGTCCCGGAG
*il10*	Forward	GACTTTAAGGGTTACCTGGGTTG
	Reverse	TCACATGCGCCTTGATGTCTG
*ccl20*	Forward	TGCTGTACCAAGAGTTTGCTC
	Reverse	CGCACACAGACAACTTTTTCTTT
**Mouse Genes**		**Primer (5′ to 3′)**
*gapdh*	Forward	AGGTCGGTGTGAACGGATTTG
	Reverse	TGTAGACCATGTAGTTGAGGTCA
*ifng*	Forward	TGCTGTACCAAGAGTTTGCTC
	Reverse	CGCACACAGACAACTTTTTCTTT
*Il17a*	Forward	TTTAACTCCCTTGGCGCAAAA
	Reverse	CTTTCCCTCCGCATTGACAC
*Il17f*	Forward	TGCTACTGTTGATGTTGGGAC
	Reverse	CAGAAATGCCCTGGTTTTGGT
*csf2*	Forward	ATCAAAGAAGCCCTGAACCT
	Reverse	GTGTTTCACAGTCCGTTTCC
*rorgt*	Forward	AGCTTTGTGCAGATCTAAGG
	Reverse	TGTCCTCCTCAGTAGGGTAG
*t-bet*	Forward	AGCAAGGACGGCGAATGTT
	Reverse	GTGGACATATAAGCGGTTCCC
*tnfa*	Forward	CCGATGGGTTGTACCTTGTC
	Reverse	CGGACTCCGCAAAGTCTAAG
*ccl2*	Forward	TTAAAAACCTGGATCGGAACCAA
	Reverse	GCATTAGCTTCAGATTTACGGGT
*Il6*	Forward	TAGTCCTTCCTACCCCAATTTCC
	Reverse	TTGGTCCTTAGCCACTCCTTC
*il8*	Forward	TGTTGAGCATGAAAAGCCTCTAT
	Reverse	AGGTCTCCCGAATTGGAAAGG
*il1b*	Forward	CTGTGACTCATGGGATGATGATG
	Reverse	CGGAGCCTGTAGTGCAGTTG
*il10*	Forward	ATAACTGCACCCACTTCCCA
	Reverse	GGGCATCACTTCTACCAGGT
*ccl20*	Forward	ATGGCCTGCGGTGGCAAGCGTCTG
	Reverse	TAGGCTGAGGAGGTTCACAGCCCT

## Data Availability

The data analyzed during the current study are available from the corresponding author on reasonable request.

## Supplementary Materials

The following supporting information can be downloaded at: https://www.mdpi.com/article/10.3390/ijms231911988/s1, Figure S1: IL-24 does not affect IL-10 expression and Treg differentiation; Figure S2: IL-24 inhibits antigen specific Th1 and Th17 responses.
