# Supplementary material for: Multifunctional Interleukin-24 Resolves Neuroretina Autoimmunity via Diverse Mechanisms
Source: Int J Mol Sci. 2022 Oct 9;23(19):11988. doi: 10.3390/ijms231911988 (PMC9570500; doi:10.3390/ijms231911988)
Supplement: Supplementary file 1 [file ijms-23-11988-s001.zip › ijms-1872326-supplementary.pdf]

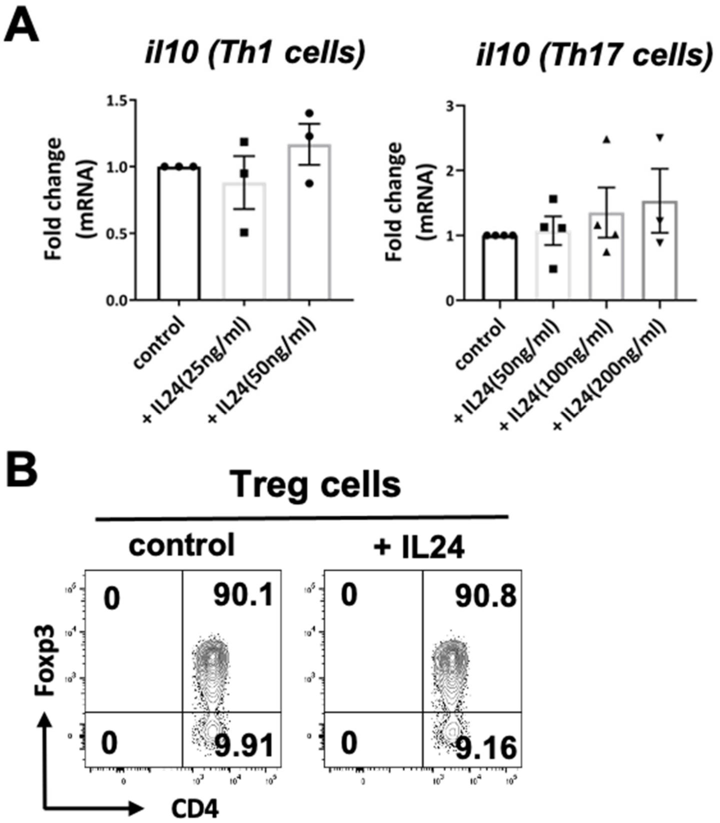

Figure S1. IL-24 did not affect IL-10 expression or Treg differentiation. CD4+CD62L+ T cells from spleens and LNs of C57BL/6 mice were isolated and polarized under Th1, Th17, or Treg conditions, with anti-CD3/CD28 antibodies for (A) 3 or (B) 4 days with and without recombinant IL-24. (A) Relative gene expression of IL-10 in Th1 and Th17 cells. Data represent means ± SEMs of (A) three or (B) four independent experiments. One-way ANOVA. (B) Representative flow cytometry Foxp3 expression in Treg cells.

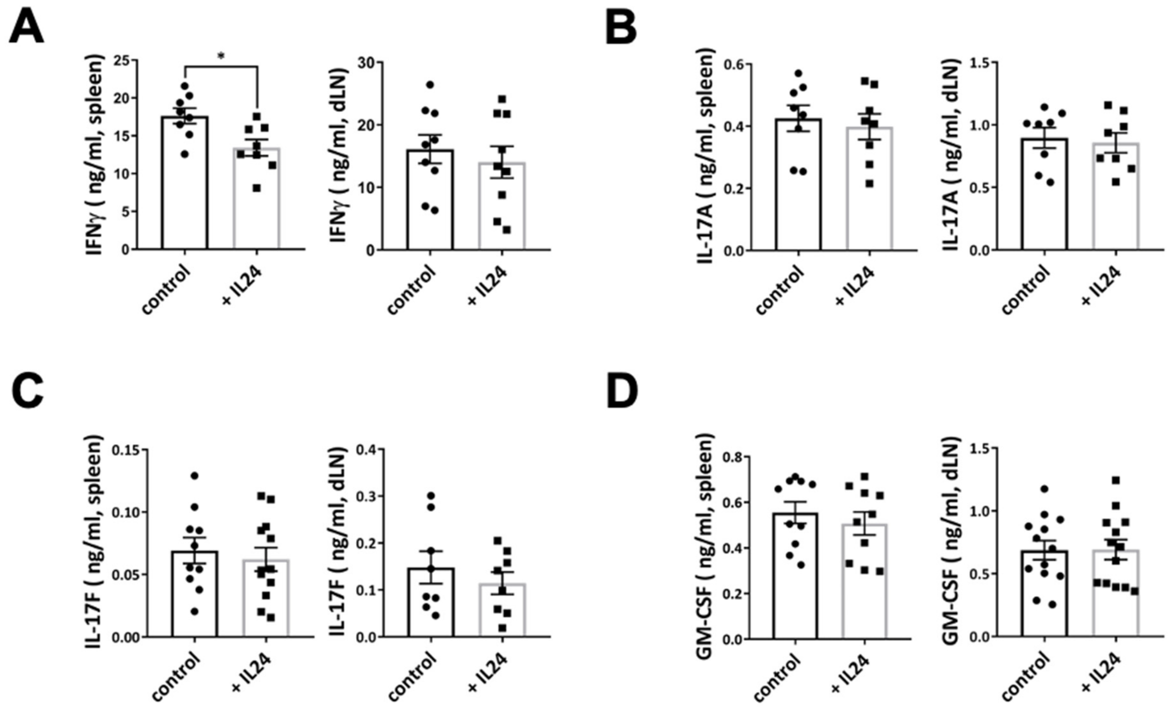

Figure S2. IL-24 inhibits antigen-specific Th1 and Th17 responses. (A-D) IRBP-specific IFN-γ, IL-17A, IL-17F, and GM-CSF production in the spleens and dLNs of EAU mice. Cells were isolated from spleens or dLNs of the EAU mice 21-24 days after immunization and were stimulated with 10 mg/mL $IRBP_{1-20}$ with and without recombinant IL-24. After 48 h, culture supernatants were collected and the cytokine production was determined with ELISA. Data represent means ± SEMs of three independent experiments. * $p < 0.05$, Student's t test.
